# Clinico-Pathological Association of Delineated miRNAs in Uveal Melanoma with Monosomy 3/Disomy 3 Chromosomal Aberrations

**DOI:** 10.1371/journal.pone.0146128

**Published:** 2016-01-26

**Authors:** Nalini Venkatesan, Jagat Kanwar, Perinkulam Ravi Deepa, Vikas Khetan, Tamsyn M. Crowley, Rajeswari Raguraman, Ganesan Sugneswari, Pukhraj Rishi, Viswanathan Natarajan, Jyotirmay Biswas, Subramanian Krishnakumar

**Affiliations:** 1 Larsen & Toubro Department of Ocular Pathology, Vision Research Foundation, Sankara Nethralaya, 18/41, College road, Chennai—600006, India; 2 Nanomedicine-Laboratory of Immunology and Molecular Biomedical Research (NLIMBR), School of Medicine (SoM), Molecular and Medical Research (MMR) Strategic Research Centre, Faculty of Health, Deakin University, Pigdons Road, Waurn Ponds, Geelong, Victoria 3217, Australia; 3 Department of Biological Sciences, Birla Institute of Technology and Science (BITS), Pilani-333031, Rajasthan, India; 4 Department of Vitreoretinal and Ocular Oncology, Medical Research Foundation, Sankara Nethralaya, 18/41, College road, Chennai—600006, India; 5 School of Medicine, Deakin University, and Australian Animal Health Laboratories, CSIRO, Australia; 6 Department of Bio-statistics, Medical Research Foundation, Sankara Nethralaya, 41, College road, Chennai—600006, India; Peking Union Medical College Hospital, CHINA

## Abstract

**Purpose:**

To correlate the differentially expressed miRNAs with clinico-pathological features in uveal melanoma (UM) tumors harbouring chromosomal 3 aberrations among South Asian Indian cohort.

**Methods:**

Based on chromosomal 3 aberration, UM (n = 86) were grouped into monosomy 3 (M3; n = 51) and disomy 3 (D3; n = 35) by chromogenic in-situ hybridisation (CISH). The clinico-pathological features were recorded. miRNA profiling was performed in formalin fixed paraffin embedded (FFPE) UM samples (n = 6) using Agilent, Human miRNA microarray, 8x15KV3 arrays. The association between miRNAs and clinico-pathological features were studied using univariate and multivariate analysis. miRNA-gene targets were predicted using Target-scan and MiRanda database. Significantly dys-regulated miRNAs were validated in FFPE UM (n = 86) and mRNAs were validated in frozen UM (n = 10) by qRT-PCR. Metastasis free-survival and miRNA expressions were analysed by Kaplen-Meier analysis in UM tissues (n = 52).

**Results:**

Unsupervised analysis revealed 585 differentially expressed miRNAs while supervised analysis demonstrated 82 miRNAs (FDR; Q = 0.0). Differential expression of 8 miRNAs: *miR-214*, miR-149*, *miR-143*, *miR-146b*, *miR-199a*, *let7b*, *miR-1238* and *miR-134* were studied. Gene target prediction revealed *SMAD4*, *WISP1*, *HIPK1*, *HDAC8* and *C-KIT* as the post-transcriptional regulators of *miR-146b*, *miR-199a*, *miR-1238* and *miR-134*. Five miRNAs (*miR-214*, *miR146b*, *miR-143*, *miR-199a* and *miR-134)* were found to be differentially expressed in M3/ D3 UM tumors. In UM patients with liver metastasis, miR-149* and *miR-134* expressions were strongly correlated.

**Conclusion:**

UM can be stratified using miRNAs from FFPE sections. miRNAs predicting liver metastasis and survival have been identified. Mechanistic linkage of de-regulated miRNA/mRNA expressions provide new insights on their role in UM progression and aggressiveness.

## Introduction

Uveal Melanoma (UM) is a primary adult intraocular tumor. The prevalence of UM is 0.02% in South Asian Indian populations [1]. According to Collaborative Ocular Melanoma Study group (COMS, 2001), about 50% of UM patients develop liver metastasis within 10–15 years of enucleation [2]. UM diagnosis and hepatic metastasis prediction is critical in prognosis and planning therapeutic regimes [3,4]. The existing prognostic factors include clinical parameters, histopathological parameters, extra-ocular extension, immune-markers, chromosomal aberrations (1, 6, 8, 13q and 16p) and germ line mutations (*BAP1* and *GNA11/ GNAQ)* [1,5,6,7].

Extensive molecular studies using gene expression and chromosomal aberration analysis have helped to stratify UM into 2 classes—class 1 tumors with low risk of liver metastasis, associated with disomy3 (D3), and class 2 tumors with high risk of liver metastasis associated with monosomy3 (M3) [8]. Unfortunately, there is a lack of accuracy in the molecular genetic testing due to intra-tumoral heterogeneity [9] and micro-deletions of genes prevailing in UM tumors decreasing the precision in identifying micro-metastases [10].

MicroRNAs (miRNAs) are non-coding RNAs that regulate the gene expression at the post-transcriptional stage and they play an important role in tumor progression and metastasis. miRNAs based biomarkers are gaining importance in cancer diagnosis and for prognosis [11,12]. There are a few earlier studies on miRNA profiles in UM. In one study, miRNA profiles in UM have been studied using primary tumor tissues, primary cells and cell lines [13,14,15]. In the second study, 6 miRNAs (*let-7b*, *miR-199a*, miR-199a*, *miR-143*, *miR-193b*, *and miR-652*) were identified to differentiate class 1 and class 2 UM tumors [13]. In the third study, prognostic significance of chromosome 3 loss and 8q gain was observed in UM archival samples, however a clear correlation was not observed between miRNA expression with metastasis and survival [16].

In India, majority of the melanomas are enucleated by the ophthalmologist and a small fraction of the patients who are treated at tertiary referral eye centre also have opportunity for plaque therapy [17]. Currently, much emphasis is laid on the largest tumor diameter (LTD) and a few centres do offer in-situ hybridisation (CISH) based M3 detection [18]. Here, we explored the potential of miRNAs as in UM with M3/D3, and their association with liver metastasis in South Asian Indian cohort of uveal melanomas. The merits of the present study are the availability of the clinico-pathological records along with the corresponding formalin-fixed paraffin embedded (FFPE) melanoma tissues for miRNA analysis. FFPE samples can be used for miRNA expression analysis as the secondary structures are not altered even after fixation processes [19].

## Materials and Methods

### Sample collection

FFPE eyeballs (n = 86) diagnosed as UM at Medical Research Foundation, Sankara Nethralaya, (2009–2013) were included in the study. Duly signed consent forms from the patient/guardian as a part of clinical management were obtained from enrolled patients. Normal melanocytes (n = 5) were collected from the human cadaveric eyeballs received at CU Shah Eye bank (http://www.sankaranethralaya.org/eye-bank.html) during 2011–2012. The study was reviewed and approved by the local ethics committee at Vision Research Foundation, Sankara Nethralaya and the committee deemed that it conformed to the principles of research, in accordance with Helsinki Declaration (Ethics number: 146b-2009-P).

### Specimen selection

#### Clinico-pathological features

Study cohort included 58 male and 28 female patients with the median age of 48.5 years. The study group consisted of 12 patients diagnosed with ciliary body melanoma and 74 with choroidal melanoma. Mean of the largest tumor diameter (LTD) was 13.7mm X 9.6mm. Haematoxylin and Eosin slides were reviewed and 1–2 representative tumor tissue blocks were selected. History of liver metastasis was obtained from patient medical records. The study included UM tumors with cell types: spindle cells (n = 27), epitheliod (n = 15) and mixed cell type (n = 44). These 86 UM tumors includes 37 UM with scleral extension; 8 UM with extension into vortex veins and 11 UM with extension into orbit. The 52 patients (60.46% corresponding to 52/86 cohort) with 2–5 years of follow-up were considered for the metastasis-free survival analysis (Kaplen-Meier). The clinical follow-up study revealed 17 patients diagnosed with liver metastasis. The clinico-pathological variables (age, sex, specimen obtained, cell type and LTD) provided as S1 Table.

#### Chromogenic in-situ hybridisation

Centromeric probes (Invitrogen, USA) were used to detect chromosome 3 aberrations and disomy 18 (control). Hybridisation and scoring protocols were followed as reported earlier [18].

#### Immunohistochemistry

Immunohistochemical detection of HSP27 on FFPE tumor sections was performed by using a rabbit polyclonal antibody (AM171-10M, Biogenex, CA, USA), Super Sensitive ^TM^ Polymer- HRP detection system (Biogenex, CA, USA) and aminoethyl carbazole according to the manufacturer’s instructions. Breast cancer cell line (MCF-7) served as positive control [20]. The immune-score ranges from 0–12 [21].

#### miRNA profiling

Three M3 and three D3 uveal melanoma tumors were taken for expression studies. The study was carried out using technical duplicates. The small RNA was extracted from the FFPE tissues using miRVANA kit (Ambion, Life Technologies, USA) following manufacturer’s protocol. The processed samples were hybridized on the Human miRNA microarray, 8x15KV3 array as per manufacturer’s instruction. The microarray slide was scanned using Agilent Scanner (Agilent Technologies, Part Number G2565CA).

#### qRT-PCR

Total RNA was isolated from FFPE tissues using Recover All^™^ total nucleic acid isolation (Ambion, Life technologies, USA) as per manufacturer’s protocol. Reverse transcription (RT) of mature miRNA in 100 ng/μl of total RNA using TaqMan MicroRNA RT kit (Applied Biosystems, Foster City, CA) was carried out following manufacturer’s protocol. For gene expression studies, RT was carried out using oligo dT random primers and Sensiscript II kit (205211, Qiagen, Santa Clara, CA). Real-time PCR was performed using 1X Universal PCR Master Mix, taqman miRNA probes (ABI Applied Biosystem, USA) and 1X Universal RT^2^ Real Time ^™^ SyBr Green/ROX PCR master Mix (Catalogue No: 330520, SABiosciences, USA) according to the manufacturer’s instructions. Description of taqman probes and primers were tabulated (S2 Table). The unit expression is log_2_ transformed ratios.

### Statistical analysis

#### qRT- PCR data analysis

The miRNA expressions were derived after normalizing with the mean expression of normal melanocytes (n = 5). Cut-offs used for de-regulation in miRNA expression was: greater than 1(log_2_ ratio) as positive expression and less than—0.5 (log_2_ ratio) as negative. Pearson’s correlation, chi-square test, independent student‘s-t test and ANOVA were used to derive the significance between the variables. Paired student’s-t test was used to derive the significance among miRNA expressions between M3/D3 tumors. Kaplen-Meier’s test was used to assess the survival rates for the presence of eight miRNAs.

#### Microarray analysis

Cut-offs used for de-regulation in miRNA expression was: greater than 1 (log_2_ transformed value) in M3 detected tumors and less than 1 (log_2_ transformed value) in chromosome 3 balanced tumors. ANOVA was used to identify the highly expressed miRNA in the M3 tumors compared with D3 tumors. Significance Analysis of Microarray (SAM) was performed to derive the significant miRNAs (Q = 0.0). Experimentally validated targets for the differentially expressed miRNAs were obtained from TargetScan, MiRanda and miRTarbase. Further, they were subjected to GO and Pathway enrichment analysis using PANTHER data base with p-value cut-off ≤ 0.05 along with Boneferroni FDR correction. Downstream effects analysis was performed to identify the biological processes and functions that are likely to be causally affected by identified regulated genes. We used the Ingenuity Pathway Analysis (IPA, Ingenuity^®^ Systems) regulation z-score algorithm to identify biological functions that are expected to be more active in UM (increased—positive z-score) and less active (decreased—negative z-score). The p-value was calculated using the Fischer's exact test and reflects the likelihood that the association between a set of genes/miRNAs in our dataset and a biological function is significant (p-value ≤ 0.05).

## Results and Discussion

### Grouping of UM into monosomy of chromosome 3 (M3) / disomy of chromosome 3 (D3)

Chromosome 3 loss was detected in 59.30% [51/86] of UM, and presence of both the copies of chromosome 3 was detected in 40.69% [35/86] UM using chromogenic in-situ hybridisation (CISH). Among the UM which had metastasized to liver (n = 17), M3 was detected in 64.7% [11/17] and D3 was detected in 35.29% [6/17] UM (S1 Fig). Here, emphasis was laid on the observation of D3 in UM that had metastasized to the liver. Existence of intra-tumoral heterogeneity in UM [9] might have contributed to this variation in metastatic prediction.

Based on an earlier report, UM were further grouped in to M3/D3 by analysing the expression of Heat shock protein 27 (HSP27) protein using immunohistochemistry [21]. S1 Table indicates the distribution of HSP27 protein expression in UM. Photomicrographs reveal the cytoplasmic positivity of HSP27 expression in UM sections (S2 Fig). A significant association was observed between HSP27 expression and M3 (p-value = 0.021) as indicated earlier [21]. Clinico-pathological descriptions of tumors, hybridization scores and immuno-scores for individual UM are tabulated in S1 Table. Their statistical significance with M3 and liver metastasis are indicated in S3 and S4 Tables. A significant association of M3 to tumor base (p = 0.002) was observed.

### Selection of significantly dys-regulated miRNAs using a high throughput analysis

The miRNAs were filtered using unsupervised and supervised data analysis. The common pool of miRNAs thus obtained was selected for validation by qRT-PCR. Data of miRNA profiling has been submitted to GEO database (GSE68828).

### Data analysis of miRNA profiling

**Fig 1:**
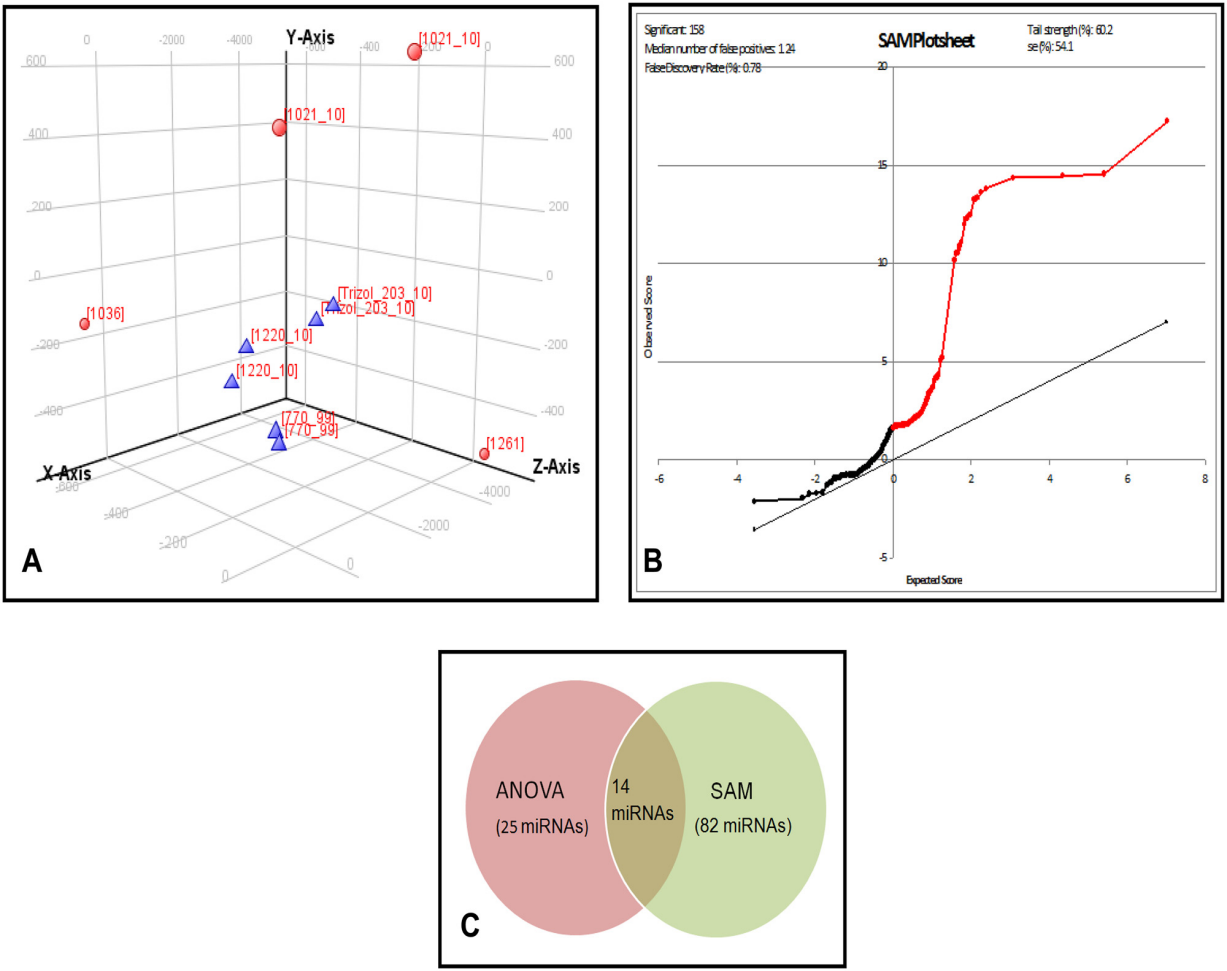
Unsupervised and supervised analysis of miRNA expression in UM. A: Graphical representation of the unsupervised analysis: principal component analysis (PCA) for the UM tumor tissues: M3 (n = 3), and D3 (n = 3) showing the clustering of samples correspondingly in to two distinct groups (M3 and D3). Blue colored triangle denotes M3 UM tumors while red colored spheres denotes D3 UM tumors. B: Supervised analysis: The significance analysis of microarray (SAM) plot reveals the de regulated miRNAs between the monosomy and disomy formalin fixed UM primary tumors. The false discovery rate (FDR) has set at Q = 0.78%. The list of miRNAs with Q = 0.0 has been considered for the validation by qRT-PCR. C: Venn diagram on intersection of miRNAs obtained from ANOVA and SAM. *miR-134*, *miR-1238*, miR-149* are the common select miRNAs observed from this study.

In unsupervised analysis, Principal Component Analysis (PCA) revealed clustering of three M3 UM tumors forming a single group and correlated with the CISH based classification. The slight difference within the D3 UM tumors could be due to the heterogeneity in the tumor infiltrating lymphocytes and the cell type (Fig 1A). The miRNA profiling of the tumors (n = 6) revealed a total of 585 differentially expressed miRNAs between M3 (n = 3) and D3 (n = 3) samples. S3 Fig shows the hierarchical cluster for the top 100 differentially expressed miRNAs with p<0.05. Data analysis using ANOVA listed 25 up-regulated miRNAs and 26 down-regulated miRNAs in M3 tumors relative to D3 tumors. (S5 Table shows top 10 miRNAs).

In supervised analysis, SAM, we observed 82 differentially expressed miRNAs with the false discovery rate (FDR); Q = 0.0 (Fig 1B). Top 10 up-regulated miRNAs with maximum score and high fold change were: *miR-317-5p*, *miR-373*, *miR-1268*, miR-191*, *miR-150*, *miR-1275*, *miR-188-5p*, *miR-1238*, *miR-134* and *miR-296-5p*.

**Fig 2:**
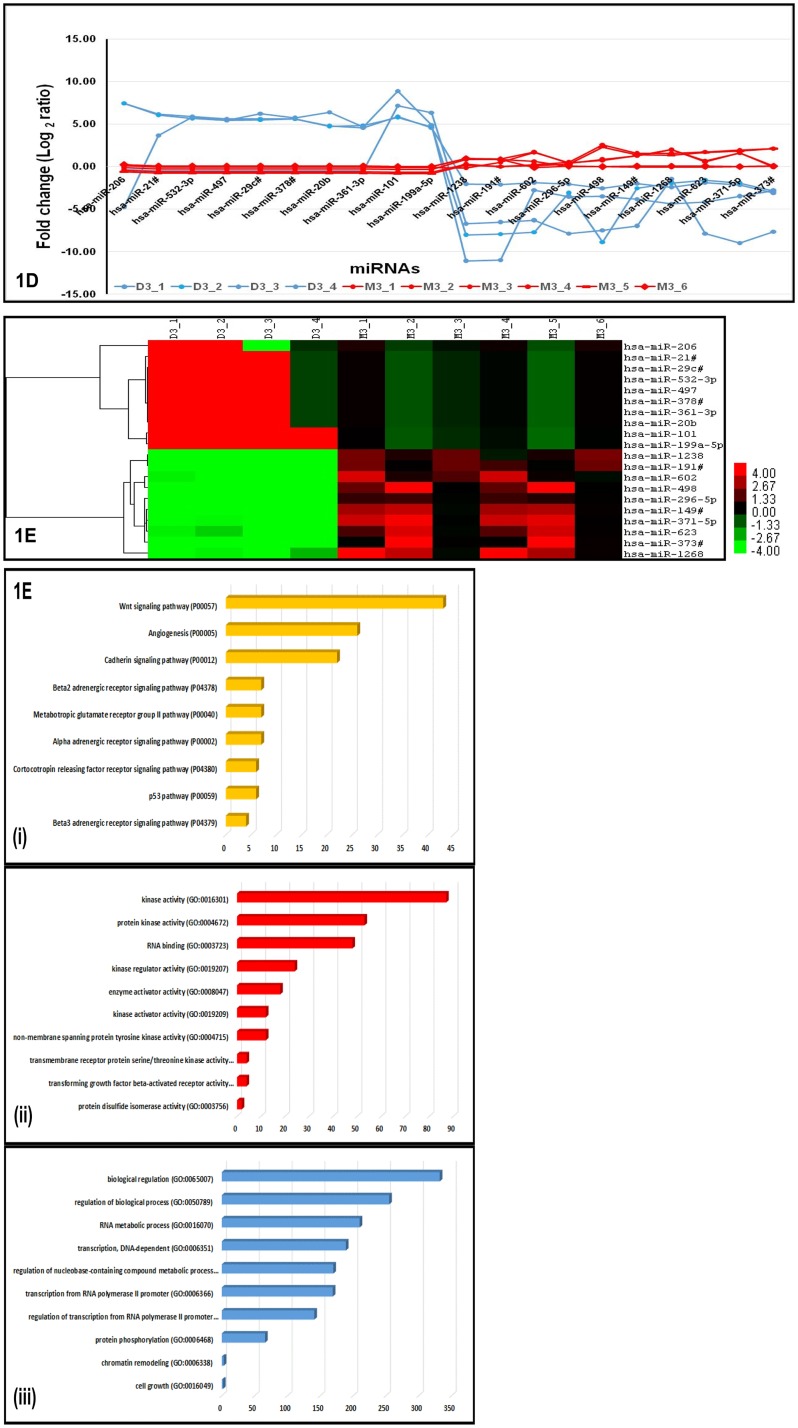
Significantly dys-regulated miRNAs and pathways in UM. A: Trendline graph shows the absolute expression of key miRNAs identified by ANOVA and SAM. The differentially expressed miRNAs indicates the discriminating profiles in M3 and D3 UM tumors. The blue line indicates the miRNA expression in D3 UM tumors while red line indicates the miRNA expression in M3 tumors. B: Hierarchical cluster shows key miRNAs subjected to unsupervised analysis. The differentially expressed miRNAs indicates the co-expression pattern across M3 and D3 UM tumors. The green colour indicates the down-regulated miRNAs while the red colour indicates the up-regulated miRNAs in M3 and D3 UM tumors. C: Analysis of key gene ontology (GO) and Pathway Enrichment by the differentially expressed miRNAs. A few of the pathways (i) and GO (ii and iii) targeted by these differentially expressed miRNAs using miRTarbase and PANTHER data base with p-value cut-off ≤0.05 along with Boneferroni FDR correction.

On intersection of miRNAs detected in both ANOVA and SAM analysis here, we observed 14 up-regulated miRNAs in common (Fig 1C). Absolute expression levels of key miRNAs identified to be differentially expressed by ANOVA and SAM methods showed (Fig 2A) discriminating profiles in M3 and D3 tumors, indicating the sensitivity of detection. Further, these key miRNAs subjected to unsupervised hierarchical clustering clearly identified miRNA clusters that could indicate co-expression pattern across M3 and D3 tumors (Fig 2B). Analysis of key gene ontology (GO) and pathways regulated by the differentially expressed miRNAs were identified by ways of genes that are validated and to be targeted by these miRNAs. Some of the GO and pathways targeted by these differentially expressed miRNAs include Wnt signaling; angiogenesis and p53 pathway (Fig 2C). Since, dys-regulation of p53 pathway has been reported in UM earlier [22], we explored the miRNAs and their targets and subjected to regulatory network modeling to understand the differential regulation of p53 pathway in M3 and D3 UM tumors.

### miRNAs expression in UM using qRT-PR

Three miRNAs: miR-149*, *miR-1238* and *miR-134* (which were in common with supervised and unsupervised data analysis; Fig 1C) and 5 miRNAs: *miR-214*, *miR-143*, *miR146b*, *miR-199a and let7b* (earlier shown as class 1/ class 2 discriminators) [13] were selected for validation. Also, the analysed expressions were associated with M3, D3 and liver metastasis in UM.

Among M3 UM with liver metastasis (n = 11), higher expressions of miR-149* (72.72%), *miR-1238* (100%), *miR-134* (100%), *miR-214* (54.54%), *miR-146b* (54.54%), *miR-199a* (100%) while moderate expression of *miR-143* (45.45%) and negative expression of *let-7b* (100%) was observed. Among M3 UM with no history of liver metastasis (n = 40), higher expressions of miR-149* (90.0%), *miR-1238* (97.5%) and *miR-134* (57.5%), *miR-214* (62.5%), *miR-146b* (67.5%), *miR-143* (65.0%), *miR-199a* (90.0%) and lower expression of *let-7b* (30.0%) were observed.

**Fig 3:**
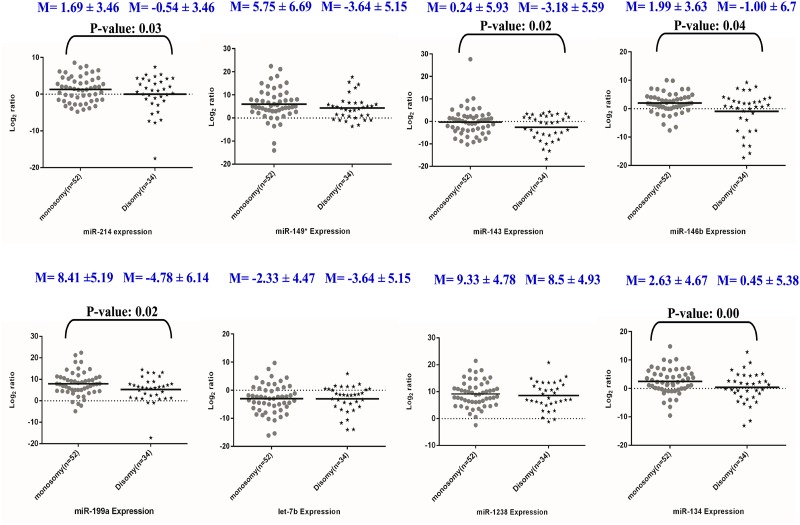
Graphical representation of the miRNA expression (fold change in log 2 ratio) in M3 / D3 UMs derived using Graph pad Prism. The dotted line represents the mean fold change of miRNA expressions while "M" denotes the mean fold change of the respective miRNA expression in UM groups.

Among D3 UM with liver metastasis (n = 6), higher expressions of miR-149* (83.3%), *miR-1238* (83.3%), *miR-199a* (100%); moderate expressions in *miR-134* (50%), *miR-214* (50.0%), while lower expressions of *miR-146b* (33.33%) and *let-7b* (25%) and negative expression in *miR-143* (100%) were observed. Among D3 UM with no liver metastasis (n = 29), higher expressions of miR-149* (72.41%), *miR-1238* (86.2%), *miR-199a* (82.75%), *miR-134* (41.37%), *miR-214* (41.37%) and *miR-146b* (58.62%), while lower expression of *miR-143* (37.93%), *let-7b* (13.79%) were observed (S6 Table and Fig 3). Relative miRNA expressions quantified by qRT-PCR in UM tumors are indicated as S7 Table.

Glycogen synthase kinase-3α (*GSK-3α*), a known gene target of miR-149* is an important melanoma growth regulator [23,24]. *MiR-1238* is known to regulate *SASH1* (SAM and SH3 domain-containing protein 1, a tumor suppressor gene), contributing to breast cancer aggressiveness [25]. Although *miR-1238* expression among the two UM groups were not significant, a higher percentage of *miR-1238* (94.18%) suggests its possible role in uveal melanoma tumorigenesis. *miR-134* is associated with invasiveness and metastasis in other cancers [26] with the putative gene targets such as *VEGFA*, *FOXM1*, *MYCN*, *CD9* and *WWOX1* genes [26,27,28]. In the present study, higher percentage of *miR-134* (94.11%) in UM with liver metastasis, irrespective of chromosome 3 aberrations, suggests it could be a potential biomarker for class 2 tumors.

Among the five miRNAs previously shown as class 2 tumor discriminators [13], four up-regulated miRNAs *(miR-214*, *miR-143*, *miR-146b and miR-199a)* showed a significant association with M3 tumors while the other miRNA, *let-7b* did not show any significant association with M3 UM (Fig 3). *miR-214* regulates cell cycle regulatory genes: phosphatase and tensin homolog (*PTEN)* [29], adipocyte protein 2 (AP2) and tumour protein 53 (TP53) genes [30]. *miR-143* is known for its oncogenic activity together with *KRAS* (Kirsten rat sarcoma viral oncogene homolog), *NF-kB* (Nuclear factor kappa B) genes in cancers [31,32]. *miR-146b*, is known for its oncogenic role in UM cells [33] with its reported gene targets *NF-kB* and *SMAD4* (Mothers against decapentaplegic Homolog 4). Deregulation of NF-*k*B pathways is known to regulate UM metastasis [34].

Interestingly, *miR-199a*, another regulator of *SMAD4* was also up-regulated in the present study (S6 Table). The tumorigenic role of this miRNA is substantiated by earlier reports in UM [13]. *Let 7b*, a known tumor suppressor miRNA is down-regulated in various cancers namely acute lymphoblastic leukaemia [35] and retinoblastoma [36]. Restoration of *let-7b* is considered as a potential therapeutic option in cancers [37]. Earlier, *let-7*, has been stated as a strong significant discriminator in primary UM [13] while let-7b* is reported at low levels in OCM1 cells [15]. Here, we observed *let-7b* de-regulation in the present UM cohort.

### Validation of target genes regulated by select miRNAs in UM

**Fig 4:**
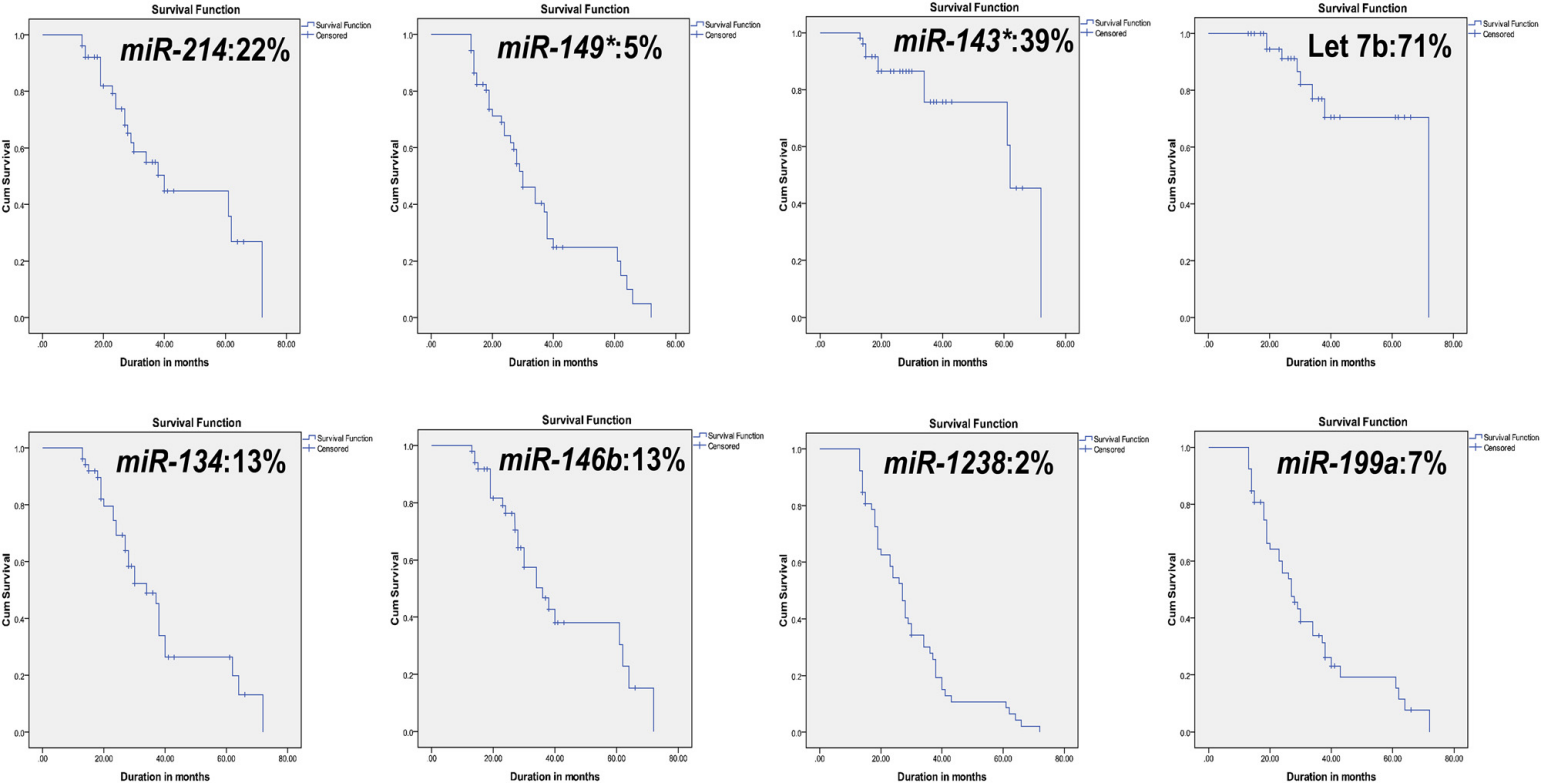
Network interaction between the dysregulated miRNAs and their gene targets in UM. A: D3 UM tumor; B: M3 UM tumor. The network is drawn with the expressions obtained by qRT-PCR using cytoscape v.2.8. Green colour indicates the down-regulation of miRNAs/ genes; red colour indicates the up-regulation of miRNAs/ genes; Yellow colour indicates varied miRNA/ gene expression.

Earlier, Harbour et al have reported a PCR based platform (DecisionDx-UM) consisting of 15 genes list to measure the risk stratification of UM tumors [4,38]. However, the additional prognostic factors specific to UM tumors such as cytogenetic analysis and miRNA expressions would confirm the uveal melanoma risk stratification [39]. Here, we have explored gene targets of select miRNAs to implicate miRNA/mRNA’s role in UM progression. Downstream analysis (S8 Table) revealed the role of up-regulated miRNAs, their regulated genes in apoptotic and cell signalling pathways. Further, gene targets prediction of the differentially expressed miRNAs revealed the negative regulation of gene lists namely (i) *SMAD4*, *WISP1*, *HDAC8 and C-KIT* by *miR-146b*, (ii) *WISP1* by *miR-1238*, *miR-134* and (iii) *SMAD4* by *miR-199a* (Fig 4). Among these interactions, we observed *SMAD4 and WISP1* as the common regulators.

*SMAD4 and WISP1* are known to regulate p53 pathway [40,41]. Infrequent mutation of p53 pathway is reported in melanoma [22]. Thus, the down-regulation of *SMAD4* gene and varied expression of *WISP1* gene (S9 Table) substantiates the varied activation of p53 pathway in UM. Here, inactivation of p53 pathway in UM was supported by the over-expression of *HIPK1* gene (S9 Table) which corroborates with an earlier report in other cancer (colorectal cancer) [42]. Further, we observed the de-regulation of *HDAC8* and *C-KIT* genes, the known targets of *miR-146b* [43]. The role of these target genes needs further investigations. Thus, the elevated level of these miRNAs together with its target genes suppression might contribute to the UM aggressiveness.

### miRNAs associated with metastasis in UM

**Fig 5:**
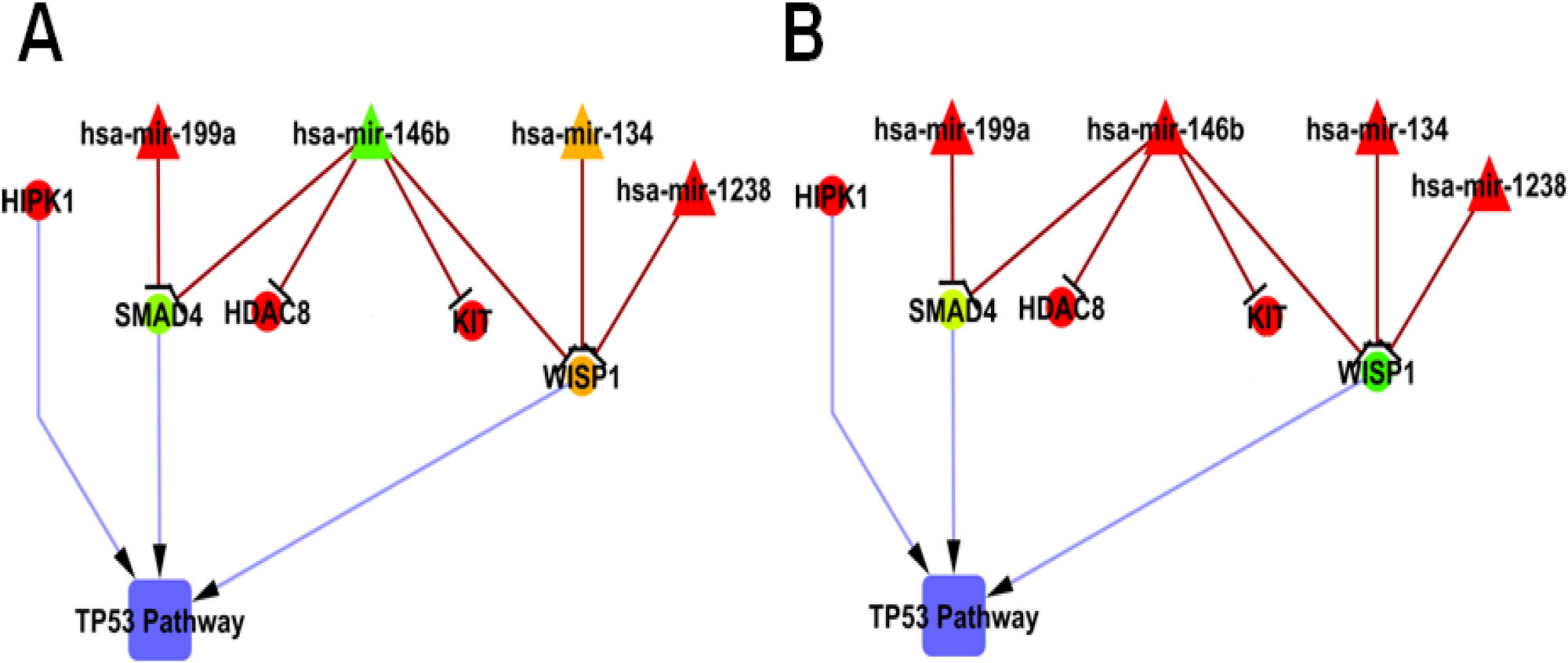
Graphical representation of metastasis-free survival was analysed by Kaplan-Meier analysis. The cumulative proportion surviving at end of the interval (60 months) is represented in percentage. Follow up of 52 patients (2–5 years of duration) were considered in the study.

Association of miRNAs with metastasis-free survival is essential for better understanding of miRNA’s involvement in UM micro-metastasis. In a very recent report by Herlihy et al (2015), de-regulation of genes coding for epigenetic modifiers have been linked with poor prognosis in UM with M3/ class 2 [44]. The data derived from metastasis-free survival analysis is presented in S6 Table and Fig 5. These results indicate that the expression of *miR-214*, miR-149*, *miR-146b*, *miR-199a*, *miR-1238 and miR-134* can be used to evaluate the metastasis-free survival in UM patients. miR-149* and *miR-134* expressions show a statistically significant association with liver metastasis.

The non-availability of 2–5 years clinical follow-up data for some patients in the cohort was a limitation in this study. We hope further studies with larger sample size and complete clinical follow-up will be able to determine the panel of miRNAs as metastatic predictors and prognostic indicators.

## Supporting Information

S1 Fig Photomicrographs of chromosome 3 abbrerations by Chromogenic in-situ hybridisation (CISH). The arrow heads indicates the hybridized spots. A: Normal retina with disomy 18 (control). B: Uveal melanoma tumour with monosomy 3, C: Uveal melanoma tumour with disomy 3.
(TIF)

S2 Fig Photomicrographs of HSP27 protein expression by Immunohistochemistry. A: Positive control, High expression of HSP27 protein in the MCF-7 cells (Breast carcinoma cell line); B: Negative control (performed by the exclusion of primary antibody), Absence of HSP27 protein in the MCF-7 cells (Breast carcinoma cell line); C & D: High to Moderate expression of HSP27 in the D3 melanoma tumors; E: Low to Negative expression of HSP27 in the M3 melanoma tumors.
(TIF)

S3 Fig Unsupervised analysis: Hierarchical cluster of the top 100 differentially expressed miRNAs between M3 and D3 UM primary tumours (FFPE). The green colour indicates the down-regulated miRNAs while red color indicates the up-regulated miRNAs. The range of the significant de-regulation is -2.0 to +2.0 log_2_ ratio.
(TIF)

S1 Table Clinico-pathological description, chromosome 3 aberration and HSP27 protein expression in UM tumor tissues. (DOC)

S2 Table Details of taqman probes and primers used in qRT-PCR. (DOC)

S3 Table Correlation analysis of Chromosome 3 aberration with clinico- pathological parameters in UM patients. (DOC)

S4 Table Correlation analysis of liver metastasis with clinico- pathological parameters in UM patients. (DOC)

S5 Table List of top 10 up-regulated miRNAs obtained by ANOVA and SAM. (DOC)

S6 Table Clinico-pathological descriptions of the tumors mean and median fold change of individual miRNAs, statistical significance (p-value ≤ 0.05) derived between the clinico-pathological parameters and mean fold change of miRNAs expression. (DOC)

S7 Table Data of miRNA expressions in UM quantified by qRT-PCR. (XLS)

S8 Table Downstream effect analysis of genes and miRNAs over-expressed in UM. (DOC)

S9 Table mRNA expression analysis by qRT-PCR. (DOC)

## References

[1] Biswas J, Kabra S, Krishnakumar S, Shanmugam MP (2004) Clinical and histopathological characteristics of uveal melanoma in Asian Indians. A study of 103 patients. Indian J Ophthalmol 52: 41–44. 15132378

[2] (COMS, 2001) Assessment of metastatic disease status at death in 435 patients with large choroidal melanoma in the Collaborative Ocular Melanoma Study (COMS): COMS report no. 15. Arch Ophthalmol 119: 670–676. 11346394 10.1001/archopht.119.5.670

[3] Field MG, Harbour JW (2014) Recent developments in prognostic and predictive testing in uveal melanoma. Curr Opin Ophthalmol 25: 234–239. 24713608 10.1097/ICU.0000000000000051 PMC4467564

[4] Harbour JW, Chen R (2013) The DecisionDx-UM Gene Expression Profile Test Provides Risk Stratification and Individualized Patient Care in Uveal Melanoma. PLoS Curr 5. 10.1371/currents.eogt.af8ba80fc776c8f1ce8f5dc485d4a618 PMC3625622 23591547

[5] Aalto Y, Eriksson L, Seregard S, Larsson O, Knuutila S (2001) Concomitant loss of chromosome 3 and whole arm losses and gains of chromosome 1, 6, or 8 in metastasizing primary uveal melanoma. Invest Ophthalmol Vis Sci 42: 313–317. 11157859

[6] Njauw CN, Kim I, Piris A, Gabree M, Taylor M, et al (2012) Germline BAP1 inactivation is preferentially associated with metastatic ocular melanoma and cutaneous-ocular melanoma families. PLoS One 7: e35295 10.1371/journal.pone.0035295 22545102 PMC3335872

[7] Van Raamsdonk CD, Griewank KG, Crosby MB, Garrido MC, Vemula S, et al (2010) Mutations in GNA11 in uveal melanoma. N Engl J Med 363: 2191–2199. 10.1056/NEJMoa1000584 21083380 PMC3107972

[8] Onken MD, Worley LA, Ehlers JP, Harbour JW (2004) Gene expression profiling in uveal melanoma reveals two molecular classes and predicts metastatic death. Cancer Res 64: 7205–7209. 15492234 10.1158/0008-5472.CAN-04-1750 PMC5407684

[9] Mensink HW, Vaarwater J, Kilic E, Naus NC, Mooy N, et al (2009) Chromosome 3 intratumor heterogeneity in uveal melanoma. Invest Ophthalmol Vis Sci 50: 500–504. 10.1167/iovs.08-2279 18824727

[10] Damato B (2012) Progress in the management of patients with uveal melanoma. The 2012 Ashton Lecture. Eye (Lond) 26: 1157–1172. 22744385 10.1038/eye.2012.126 PMC3443832

[11] Cheng G (2015) Circulating miRNAs: roles in cancer diagnosis, prognosis and therapy. Adv Drug Deliv Rev 81: 75–93. 10.1016/j.addr.2014.09.001 25220354

[12] Ferracin M, Veronese A, Negrini M (2010) Micromarkers: miRNAs in cancer diagnosis and prognosis. Expert Rev Mol Diagn 10: 297–308. 10.1586/erm.10.11 20370587

[13] Worley LA, Long MD, Onken MD, Harbour JW (2008) Micro-RNAs associated with metastasis in uveal melanoma identified by multiplexed microarray profiling. Melanoma Res 18: 184–190. 18477892 10.1097/CMR.0b013e3282feeac6

[14] Yang C, Wei W (2011) The miRNA expression profile of the uveal melanoma. Sci China Life Sci 54: 351–358. 10.1007/s11427-011-4149-y 21509659

[15] Venza M, Dell'Aversana C, Visalli M, Altucci L, Teti D, et al (2014) Identification of microRNA expression patterns in cutaneous and uveal melanoma cell lines. Tumori 100: e4–7. 24675509 10.1700/1430.15828

[16] Larsen AC, Holst L, Kaczkowski B, Andersen MT, Manfe V, et al (2014) MicroRNA expression analysis and Multiplex ligation-dependent probe amplification in metastatic and non-metastatic uveal melanoma. Acta Ophthalmol 92: 541–549. 10.1111/aos.12322 24373459

[17] Khetan V, Gopal L, Shanmugam MP, Gupta A, Sharma T, et al (2014) Brachytherapy of intra ocular tumors using 'BARC I-125 Ocu-Prosta seeds': an Indian experience. Indian J Ophthalmol 62: 158–162. 10.4103/0301-4738.128618 24618486 PMC4005230

[18] Radhakrishnan A, Badhrinarayanan N, Biswas J, Krishnakumar S (2009) Analysis of chromosomal aberration (1, 3, and 8) and association of microRNAs in uveal melanoma. Mol Vis 15: 2146–2154. 19898689 PMC2773734

[19] Xi Y, Nakajima G, Gavin E, Morris CG, Kudo K, et al (2007) Systematic analysis of microRNA expression of RNA extracted from fresh frozen and formalin-fixed paraffin-embedded samples. RNA 13: 1668–1674. 17698639 10.1261/rna.642907 PMC1986820

[20] Ciocca DR, Calderwood SK (2005) Heat shock proteins in cancer: diagnostic, prognostic, predictive, and treatment implications. Cell Stress Chaperones 10: 86–103. 16038406 10.1379/CSC-99r.1 PMC1176476

[21] Jmor F, Kalirai H, Taktak A, Damato B, Coupland SE (2012) HSP-27 protein expression in uveal melanoma: correlation with predicted survival. Acta Ophthalmol 90: 534–539. 10.1111/j.1755-3768.2010.02038.x 21114636

[22] Brantley MA, Harbour JW Jr. (2000) Deregulation of the Rb and p53 pathways in uveal melanoma. Am J Pathol 157: 1795–1801. 11106551 10.1016/s0002-9440(10)64817-1 PMC1885790

[23] Damsky WE, Curley DP, Santhanakrishnan M, Rosenbaum LE, Platt JT, et al (2011) beta-catenin signaling controls metastasis in Braf-activated Pten-deficient melanomas. Cancer Cell 20: 741–754. 10.1016/j.ccr.2011.10.030 22172720 PMC3241928

[24] Madhunapantula SV, Sharma A, Gowda R, Robertson GP (2013) Identification of glycogen synthase kinase 3alpha as a therapeutic target in melanoma. Pigment Cell Melanoma Res 26: 886–899. 10.1111/pcmr.12156 24034838 PMC4010947

[25] Zeller C, Hinzmann B, Seitz S, Prokoph H, Burkhard-Goettges E, et al (2003) SASH1: a candidate tumor suppressor gene on chromosome 6q24.3 is downregulated in breast cancer. Oncogene 22: 2972–2983. 12771949 10.1038/sj.onc.1206474

[26] Liu CJ, Shen WG, Peng SY, Cheng HW, Kao SY, et al (2014) miR-134 induces oncogenicity and metastasis in head and neck carcinoma through targeting WWOX gene. Int J Cancer 134: 811–821. 10.1002/ijc.28358 23824713

[27] el Filali M, Missotten GS, Maat W, Ly LV, Luyten GP, et al (2010) Regulation of VEGF-A in uveal melanoma. Invest Ophthalmol Vis Sci 51: 2329–2337. 10.1167/iovs.09-4739 20042655

[28] Si Z, Hersey P (1993) Expression of the neuroglandular antigen and analogues in melanoma. CD9 expression appears inversely related to metastatic potential of melanoma. Int J Cancer 54: 37–43. 8478146 10.1002/ijc.2910540107

[29] Yang H, Kong W, He L, Zhao JJ, O'Donnell JD, et al (2008) MicroRNA expression profiling in human ovarian cancer: miR-214 induces cell survival and cisplatin resistance by targeting PTEN. Cancer Res 68: 425–433. 10.1158/0008-5472.CAN-07-2488 18199536

[30] Xu CX, Xu M, Tan L, Yang H, Permuth-Wey J, et al (2012) MicroRNA miR-214 regulates ovarian cancer cell stemness by targeting p53/Nanog. J Biol Chem 287: 34970–34978. 10.1074/jbc.M112.374611 22927443 PMC3471722

[31] Zhang X, Liu S, Hu T, He Y, Sun S (2009) Up-regulated microRNA-143 transcribed by nuclear factor kappa B enhances hepatocarcinoma metastasis by repressing fibronectin expression. Hepatology 50: 490–499. 10.1002/hep.23008 19472311

[32] Xu B, Niu X, Zhang X, Tao J, Wu D, et al (2011) miR-143 decreases prostate cancer cells proliferation and migration and enhances their sensitivity to docetaxel through suppression of KRAS. Mol Cell Biochem 350: 207–213. 10.1007/s11010-010-0700-6 21197560

[33] Ventura BV, Quezada C, Maloney SC, Fernandes BF, Antecka E, et al (2014) Expression of the metastasis suppressor BRMS1 in uveal melanoma. Ecancermedicalscience 8: 410 10.3332/ecancer.2014.410 24688598 PMC3963706

[34] Dror R, Lederman M, Umezawa K, Barak V, Pe'er J, et al (2010) Characterizing the involvement of the nuclear factor-kappa B (NF kappa B) transcription factor in uveal melanoma. Invest Ophthalmol Vis Sci 51: 1811–1816. 10.1167/iovs.09-3392 19892878

[35] Mi S, Lu J, Sun M, Li Z, Zhang H, et al (2007) MicroRNA expression signatures accurately discriminate acute lymphoblastic leukemia from acute myeloid leukemia. Proc Natl Acad Sci U S A 104: 19971–19976. 18056805 10.1073/pnas.0709313104 PMC2148407

[36] Danda R, Krishnan G, Ganapathy K, Krishnan UM, Vikas K, et al (2013) Targeted expression of suicide gene by tissue-specific promoter and microRNA regulation for cancer gene therapy. PLoS One 8: e83398 10.1371/journal.pone.0083398 24391761 PMC3877029

[37] Li Y, VandenBoom TG 2nd, Kong D, Wang Z, Ali S, et al (2009) Up-regulation of miR-200 and let-7 by natural agents leads to the reversal of epithelial-to-mesenchymal transition in gemcitabine-resistant pancreatic cancer cells. Cancer Res 69: 6704–6712. 10.1158/0008-5472.CAN-09-1298 19654291 PMC2727571

[38] Harbour JW (2014) A prognostic test to predict the risk of metastasis in uveal melanoma based on a 15-gene expression profile. Methods Mol Biol 1102: 427–440. 10.1007/978-1-62703-727-3_22 24258991 PMC4476294

[39] Klufas MA, Itty S, McCannel CA, Glasgow BJ, Moreno C, et al (2015) Variable Results for Uveal Melanoma-Specific Gene Expression Profile Prognostic Test in Choroidal Metastasis. JAMA Ophthalmol 133: 1073–1076. 10.1001/jamaophthalmol.2015.1790 26086628

[40] Wang CL, Wan YL, Liu YC, Huang ZQ (2006) TGF-beta1/SMAD signaling pathway mediates p53-dependent apoptosis in hepatoma cell lines. Chin Med Sci J 21: 33–35. 16615282

[41] Su F, Overholtzer M, Besser D, Levine AJ (2002) WISP-1 attenuates p53-mediated apoptosis in response to DNA damage through activation of the Akt kinase. Genes Dev 16: 46–57. 11782444 10.1101/gad.942902 PMC155313

[42] Rey C, Soubeyran I, Mahouche I, Pedeboscq S, Bessede A, et al (2013) HIPK1 drives p53 activation to limit colorectal cancer cell growth. Cell Cycle 12: 1879–1891. 10.4161/cc.24927 23676219 PMC3735702

[43] Witt O, Deubzer HE, Milde T, Oehme I (2009) HDAC family: What are the cancer relevant targets? Cancer Lett 277: 8–21. 10.1016/j.canlet.2008.08.016 18824292

[44] Herlihy N, Dogrusoz M, van Essen TH, Harbour JW, van der Velden PA, et al (2015) Skewed expression of the genes encoding epigenetic modifiers in high-risk uveal melanoma. Invest Ophthalmol Vis Sci 56: 1447–1458. 10.1167/iovs.14-15250 25593028 PMC5102441

