# Supplementary figures and images for: Clinico-Pathological Association of Delineated miRNAs in Uveal Melanoma with Monosomy 3/Disomy 3 Chromosomal Aberrations
Source: PLoS One. 2016 Jan 26;11(1):e0146128. doi: 10.1371/journal.pone.0146128 (PMC4728065; doi:10.1371/journal.pone.0146128)

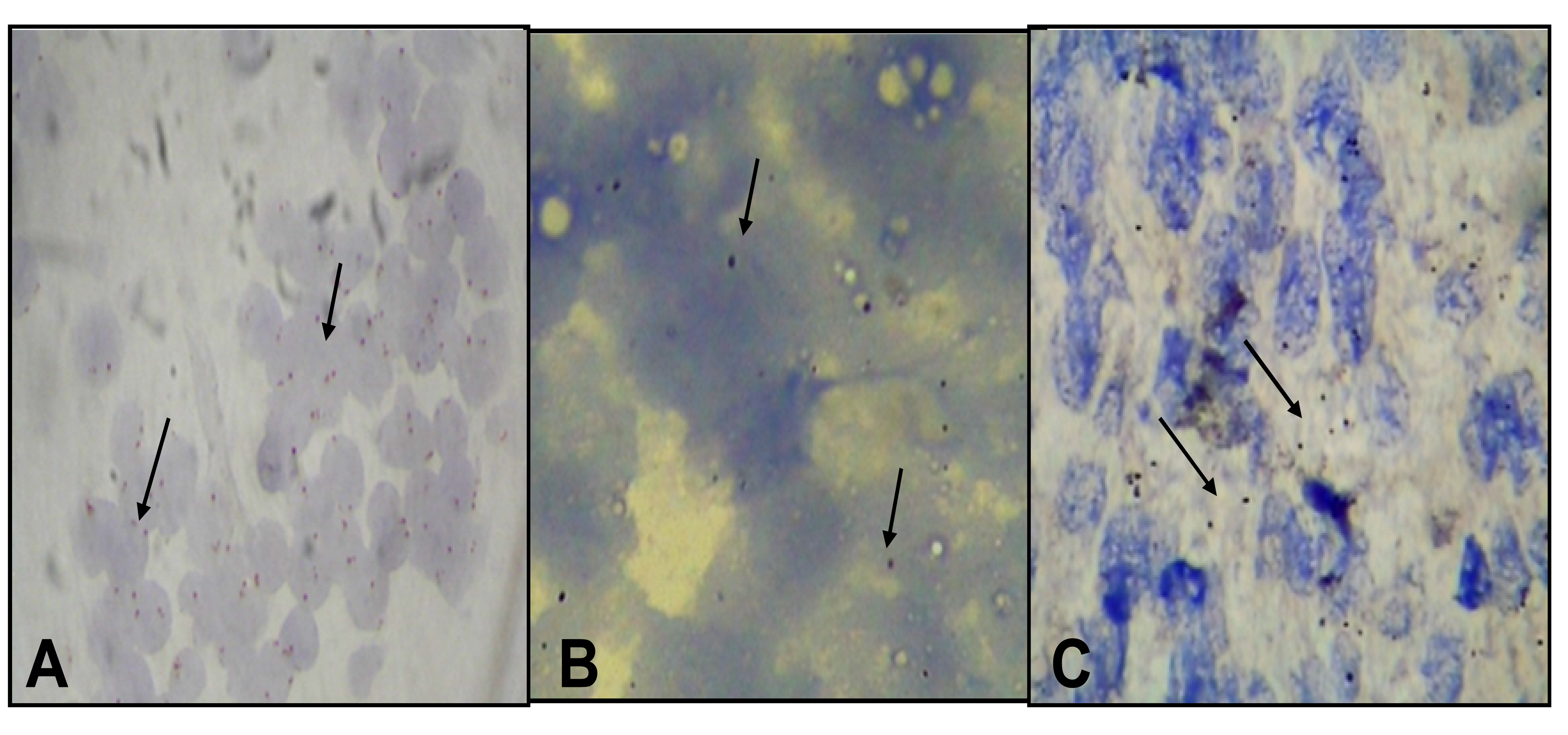

Supplement: S1 Fig — The arrow heads indicates the hybridized spots. A: Normal retina with disomy 18 (control). B: Uveal melanoma tumour with monosomy 3, C: Uveal melanoma tumour with disomy 3. (TIF) [file pone.0146128.s001.tif]

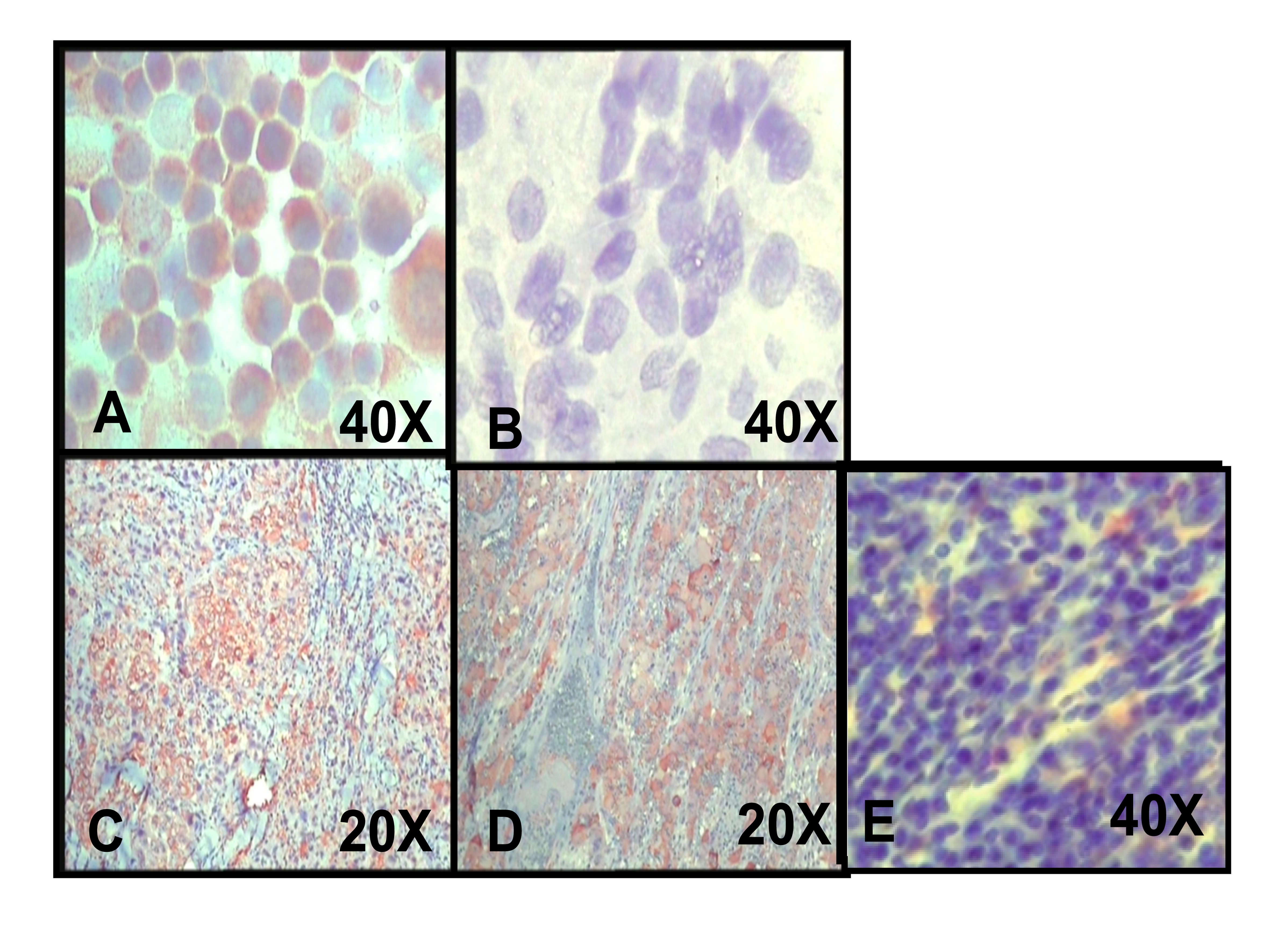

Supplement: S2 Fig — A: Positive control, High expression of HSP27 protein in the MCF-7 cells (Breast carcinoma cell line); B: Negative control (performed by the exclusion of primary antibody), Absence of HSP27 protein in the MCF-7 cells (Breast carcinoma cell line); C & D: High to Moderate expression of HSP27 in the D3 melanoma tumors; E: Low to Negative expression of HSP27 in the M3 melanoma tumors. (TIF) [file pone.0146128.s002.tif]

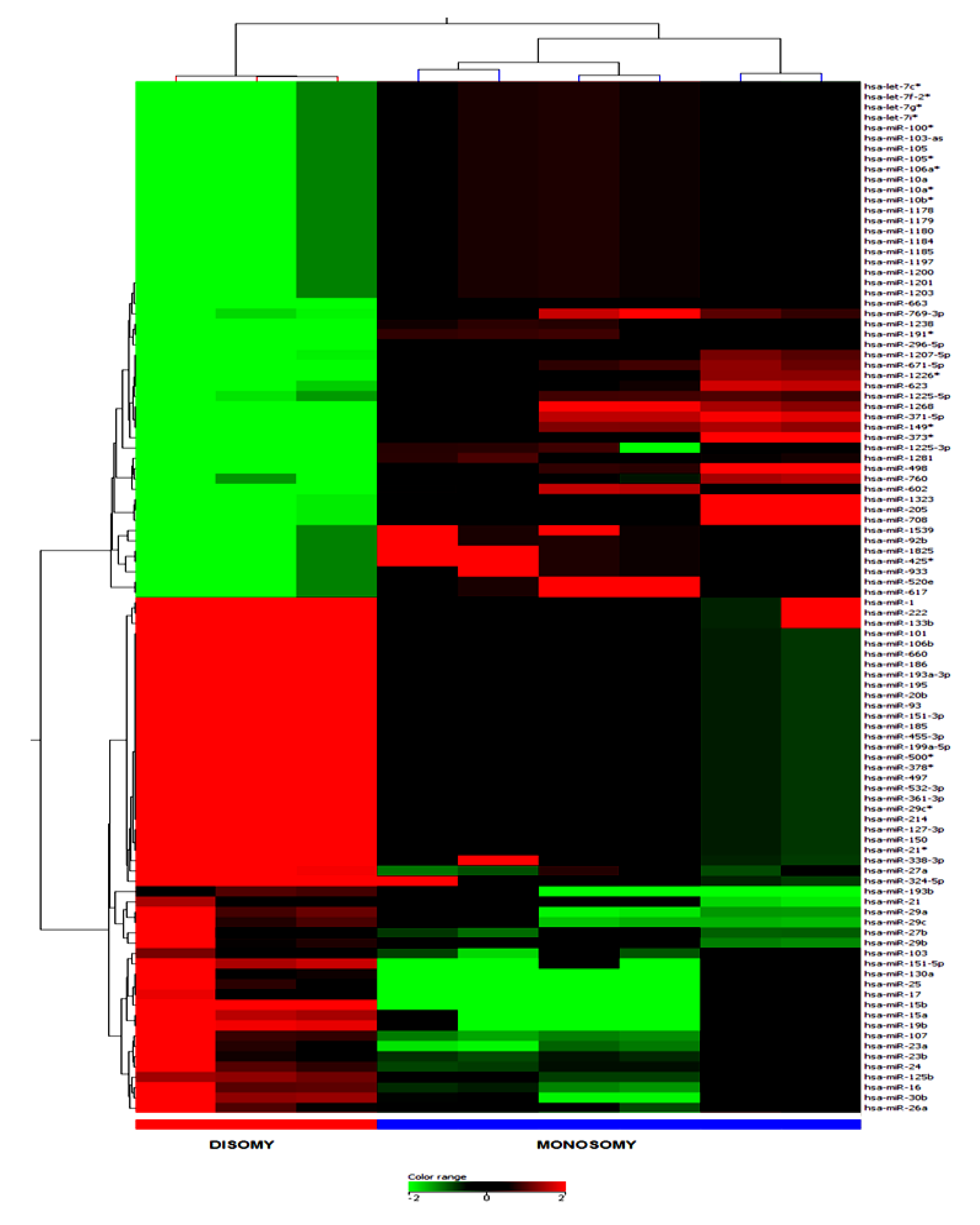

Supplement: S3 Fig — The green colour indicates the down-regulated miRNAs while red color indicates the up-regulated miRNAs. The range of the significant de-regulation is -2.0 to +2.0 log2 ratio. (TIF) [file pone.0146128.s003.tif]
