# Supplementary material for: Clinico-Pathological Association of Delineated miRNAs in Uveal Melanoma with Monosomy 3/Disomy 3 Chromosomal Aberrations
Source: PLoS One. 2016 Jan 26;11(1):e0146128. doi: 10.1371/journal.pone.0146128 (PMC4728065; doi:10.1371/journal.pone.0146128)
Supplement: S1 Table — (DOC) [file pone.0146128.s004.doc]

**S1 Table:** Clinic-pathological description, results of the In-situ hybridization and HSP27 expression for uveal melanoma tumor tissues.

| Patient ID | Age/  Sex | Ciliary body | Tumor Base (mm) | Tumor thickness (mm) | Specimen | Cell type | Chromosomal aberration (CISH) | HSP27 expression (IHC) | | | Doing Well / Metastasis |
| --- | --- | --- | --- | --- | --- | --- | --- | --- | --- | --- | --- |
|  |  |  |  |  |  |  |  | Positivity | Intensity | Immuno-score |  |
| M1 | 36/F | NO | 16.5 | 14 | OS:EB | M | M | 2 | 2 | 4 | DW |
| M2 | 57/M | NO | 9 | 9.5 | OD:EB | M | D | 2 | 2 | 4 | Metastasis |
| M3 | 36/F | NO | 10 | 11 | OD:EB | M | D | 1 | 1 | 1 | DW |
| M4 | 73/M | NO | 11.5 | 17 | OD:EB | M | D | 2 | 1 | 2 | DW |
| M5 | 47/M | NO | 14 | 9 | OS:EB | S | D | 3 | 3 | 9 | DW |
| M6 | 48/F | NO | 13.5 | 13.5 | OS:EB | E | M | 1 | 1 | 1 | DW |
| M7 | 57/M | NO | 13.4 | 14.9 | OD:EB | S | M | 1 | 1 | 1 | DW |
| M8 | 48/F | NO | 12.1 | 9.5 | OS:EB | M | M | 4 | 3 | 12 | DW |
| M9 | 51/M | YES | 1 | 2 | OD:EB | S | D | 2 | 2 | 4 | DW |
| M10 | 65/M | NO | 5.5 | 11.5 | OS:EB | E | M | 3 | 3 | 9 | DW |
| M11 | 21/M | NO | 155 | 15 | OS: Orbit | E | M | 3 | 3 | 9 | DW |
| M12 | 38/M | YES | 9 | 9.5 | OS:EB | S | M | 3 | 3 | 9 | DW |
| M13 | 61/M | NO | 14.5 | 13.5 | OD:EB | S | M | 1 | 1 | 1 | DW |
| M14 | 46/M | YES | 10 | 10 | OS:EB | M | D | 1 | 1 | 1 | DW |
| M15 | 60/F | NO | 10.5 | 12.9 | OS:EB | M | M | 2 | 1 | 2 | DW |
| M16 | 43/F | NO | 12 | 9 | OD:EB | M | M | 0 | 0 | 0 | DW |
| M17 | 17/M | NO | 7 | 4 | OD:EB | S | D | 1 | 1 | 1 | DW |
| M18 | 63/M | NO | 15 | 15 | OS:EB | M | D | 1 | 1 | 1 | DW |
| M19 | 35/F | NO | 9 | 8.5 | OD:EB | M | D | 2 | 2 | 4 | DW |
| M20 | 46/M | NO | 7 | 8 | OS:EB | M | D | 1 | 1 | 1 | DW |
| M21 | 32/F | NO | 11.5 | 15 | OD:EB | M | M | 2 | 2 | 4 | DW |
| M22 | 60/M | YES | 20 | 11.5 | OS:EB | M | M | 1 | 0 | 0 | DW |
| M23 | 62/F | NO | 11.5 | 7 | OD:EB | M | D | 1 | 1 | 1 | DW |
| M24 | 50/M | NO | 13 | 4 | OD:EB | E | M | 3 | 3 | 9 | DW |
| M25 | 23/F | NO | 10 | 8 | OD:EB | S | D | 3 | 3 | 9 | DW |
| M26 | 58/M | NO | 14 | 9 | OD:EB | S | D | 3 | 3 | 9 | DW |
| M27 | 36/M | NO | 9 | 9.5 | OD : Ex | M | M | 3 | 3 | 9 | DW |
| M28 | 51/M | NO | 16.5 | 15 | OD:EB | E | M | 4 | 3 | 12 | DW |
| M29 | 58/M | NO | 9.5 | 14 | OD:EB | M | D | 1 | 1 | 1 | DW |
| M30 | 65/F | NO | 7 | 7 | OS:EB | M | M | 4 | 3 | 12 | Metastasis |
| M31 | 70/M | NO | 6 | 10 | OS:EB | M | D | 1 | 1 | 1 | DW |
| M32 | 43/M | NO | 20 | 12 | OS:EB | M | D | 1 | 1 | 1 | DW |
| M33 | 53/F | NO | 5 | 8 | OD:EB | M | M | 3 | 3 | 9 | DW |
| M34 | 37/F | NO | 12 | 10 | OD:EB | S | D | 4 | 3 | 12 | DW |
| M35 | 33/M | NO | 12 | 8 | OS:EB | S | D | 1 | 1 | 1 | DW |
| M36 | 55/M | NO | 12 | 7 | OS:EB | S | D | 2 | 2 | 4 | DW |
| M37 | 76/M | NO | 12 | 9 | OD:EB | M | M | 1 | 1 | 1 | DW |
| M38 | 46/F | YES | 12 | 10 | OS:EB | E | D | 0 | 0 | 0 | DW |
| M39 | 63/M | NO | 7 | 4 | OD:EB | S | D | 2 | 2 | 4 | Metastasis |
| M40 | 45/F | NO | 14 | 16 | OD:EB | M | D | 2 | 2 | 4 | Metastasis |
| M41 | 41/F | NO | 20 | 20 | OS:EB | M | M | 2 | 2 | 4 | Metastasis |
| M42 | 21/M | NO | 16.5 | 12 | OS:EB | S | M | 1 | 1 | 1 | Metastasis |
| M43 | 56/M | NO | 12 | 7 | OS:EB | S | D | 3 | 3 | 9 | Metastasis |
| M44 | 30/M | YES | 14.5 | 3.5 | OS:EB | S | M | 3 | 3 | 9 | Metastasis |
| M45 | 38/M | NO | 5.5 | 11 | OS:EB | S | M | 3 | 3 | 9 | DW |
| M46 | 56/F | NO | 15 | 1 | OS: Ex | E | M | 0 | 0 | 0 | DW |
| M47 | 34/M | NO | 15 | 1 | OS: Ex | M | M | 1 | 1 | 1 | Metastasis |
| M48 | 55/M | NO | 15 | 1 | OS: Ex | E | D | 2 | 1 | 2 | Metastasis |
| M49 | 48/M | NO | 5 | 12 | OD:EB | E | M | 3 | 2 | 6 | DW |
| M50 | 28/M | NO | 15 | 1 | OD: Orbit | E | M | 2 | 1 | 2 | DW |
| M51 | 32/M | NO | 15 | 17 | OS:EB | E | D | 2 | 2 | 4 | DW |
| M52 | 26/M | NO | 10 | 0.1 | OS: Ex | E | M | 2 | 3 | 6 | DW |
| M53 | 37/F | NO | 14 | 10 | OD:EB | S | M | 1 | 1 | 1 | DW |
| M54 | 74/M | NO | 9 | 9.5 | OS:EB | M | M | 2 | 2 | 4 | DW |
| M55 | 34/M | YES | 15 | 12 | OD:EB | M | M | 2 | 2 | 4 | DW |
| M56 | 58/F | NO | 15 | 7 | OD: Ex | M | M | 1 | 2 | 2 | Metastasis |
| M57 | 28/M | NO | 12.5 | 8 | OD:EB | S | M | 1 | 2 | 2 | DW |
| M58 | 68/M | NO | 11 | 20 | OS:EB | E | M | 1 | 1 | 1 | DW |
| M59 | 54/M | NO | 17 | 14 | OS:EB | S | M | 2 | 2 | 4 | DW |
| M60 | 35/F | NO | 10 | 7 | OD: Ex | E | M | 2 | 2 | 4 | DW |
| M61 | 70/M | NO | 15 | 7 | OD: Orbit | M | M | 3 | 3 | 9 | DW |
| M62 | 44/F | NO | 12 | 7 | OS:EB | M | M | 2 | 2 | 4 | DW |
| M63 | 43/M | NO | 14 | 13 | OS:EB | S | M | 3 | 3 | 9 | Metastasis |
| M64 | 34/M | NO | 15 | 7 | OS; Ex | M | D | 1 | 1 | 1 | Metastasis |
| M65 | 55/F | NO | 9 | 9.5 | OD: Orbit | E | M | 1 | 1 | 1 | Metastasis |
| M66 | 62/M | YES | 9 | 8 | OS:EB | S | D | 3 | 2 | 6 | DW |
| M67 | 52/M | NO | 11 | 7 | OD:EB | M | M | 2 | 2 | 4 | DW |
| M68 | 46/F | NO | 6 | 5 | OD:EB | S | M | 3 | 2 | 6 | Metastasis |
| M69 | 56/M | NO | 15 | 10 | OS:EB | M | M | 1 | 1 | 1 | DW |
| M70 | 48/M | NO | 7 | 4 | OD:EB | M | D | 1 | 1 | 1 | DW |
| M71 | 74/M | NO | 15 | 8 | OS:EB | S | M | 3 | 3 | 9 | DW |
| M72 | 49/F | NO | 10 | 12 | OS:EB | M | M | 1 | 1 | 1 | DW |
| M73 | 2/F | YES | 17 | 19 | OS:EB | M | M | 1 | 1 | 1 | DW |
| M74 | 36/F | NO | 10.0 , 20.0 | 5.0 , 10.0 | OS:EB | M | D | 2 | 2 | 4 | DW |
| M75 | 60/M | NO | 14 | 11.5 | OS:EB | M | M | 3 | 3 | 9 | DW |
| M76 | 40/F | NO | 11.5 | 15 | OS:EB | S | M | 0 | 0 | 0 | DW |
| M77 | 65/M | NO | 10 | 9 | OS:EB | S | M | 0 | 0 | 0 | DW |
| M78 | 63/F | NO | 11 | 8 | OS:EB | E | D | 1 | 1 | 1 | DW |
| M79 | 52/M | NO | 15 | 10 | OS:EB | M | M | 2 | 1 | 2 | DW |
| M80 | 21/M | NO | 12 | 8 | OD:EB | M | D | 2 | 1 | 2 | DW |
| M81 | 54/M | NO | 9 | 9.5 | OS: Orbit | E | D | 4 | 3 | 12 | DW |
| M82 | 60/M | YES | 16 | 10 | OS:EB | M | D | 3 | 2 | 6 | DW |
| M83 | 51/M | NO | 9.5 | 4 | OD:EB | M | M | 3 | 2 | 6 | Metastasis |
| M84 | 35/F | NO | 15 | 7 | OD:EB | E | D | 1 | 1 | 1 | DW |
| M85 | 50/F | No | 15.0 | 6.0 | OS:EB | S | D | 2 | 2 | 4 | DW |
| M86 | 65/F | YES | 15.0 | 16.0 | OS:EB | M | M | 1 | 1 | 1 | Metastasis |

M: Male; F: Female, OS: Left eye, OD: Right eye, EB: Enucleated eyeball; Ex: Exenterated tissue; DW: Doing well and Metastasis: Liver metastasis observed during the clinical follow-up.
