# Supplementary material for: Clinico-Pathological Association of Delineated miRNAs in Uveal Melanoma with Monosomy 3/Disomy 3 Chromosomal Aberrations
Source: PLoS One. 2016 Jan 26;11(1):e0146128. doi: 10.1371/journal.pone.0146128 (PMC4728065; doi:10.1371/journal.pone.0146128)
Supplement: S2 Table — (DOC) [file pone.0146128.s005.doc]

**S2 Table**: Descriptions of taqman probes and primers used in qRT-PCR.

| **A. miRNA expression studies** | | | |
| --- | --- | --- | --- |
| **S.No** | **Assay ID** | **Name of miRNA** | **miRNA Sequence** |
|  | 000517 | *hsa-miR-214* | ACAGCAGGCACAGACAGGCAG |
|  | 001097 | *hsa-miR-146b* | UGAGAACUGAAUUCCAUAGGCU |
|  | 000466 | *hsa-miR-143** | UGAGAUGAAGCACUGUAGCUCA |
|  | 002164 | *hsa-miR-149** | AGGGAGGGACGGGGGCUGUGC |
|  | 000498 | *hsa-miR-199a* | CCCAGUGUUCAGACUACCUGUUC |
|  | 000378 | *hsa-let-7b* | UGAGGUAGUAGGUUGUGUGGUU |
|  | 002927 | *hsa-miR-1238* | CUUCCUCGUCUGUCUGCCCC |
|  | 000459 | *hsa-miR-134* | UGUGACUGGUUGACCAGAGGG |
|  | 001093 | *RU6* | CGCAAGGAUGACACGCAAAUUCGUGAAGCGUUCCAUAUUUUU |
|  | 001006 | *RNU48* | GAUGACCCCAGGUAACUCUGAGUGUGUCGCUGAUGCCAUCACCGCAGCGCUCUGACC |
| **B. Gene expression studies** | | | |
|  | *SMAD4* | | FP 5’GTTCAGGTAGGAGAGACGTTTAAGGT3’  RP 5’CCTTTACATTCCAACTGCACTCCT3’ |
|  | *c-KIT* | | FP 5′-CAGGCAACGTTGACTATCAGT-3′  RP 5′-ATTCTCAGACTTGGGATAATC-3′ |
|  | *HIPK2* | | FP 5’GCCGAGAGCGGAGACACA3’  RP 5’CTCAGCCTCAGTGGGAATCTG3’ |
|  | *WISP1* | | FP 5′-AGAGCCGC CTCTGCAACTT-3′  RP 5′-GGAGAAGCCAAGCCCATCA-3′ |
|  | *HDAC8* | | FP 5′- TGGGCAGTCGCTGGT -3′  RP 5′- GTGGCTGGGCAGTCATAA-3′ |
|  | *GAPDH* | | FP 5’-AGAAGGCTGGGGCTCATTTG-3’  RP 5’-AGGGGCCATCCACAGTCTTC-3’ |
