# Supplementary material for: Clinico-Pathological Association of Delineated miRNAs in Uveal Melanoma with Monosomy 3/Disomy 3 Chromosomal Aberrations
Source: PLoS One. 2016 Jan 26;11(1):e0146128. doi: 10.1371/journal.pone.0146128 (PMC4728065; doi:10.1371/journal.pone.0146128)
Supplement: S3 Table — (DOC) [file pone.0146128.s006.doc]

**S3 Table:** Correlation analysis of Chromosome 3 aberration with clinico pathological parameters in UM patients under study

| Clinico pathological parameters  (Independent-t –test) | p-value |
| --- | --- |
| HSP27 | 0.021 |
| Age | 0.17 |
| Sex | 0.39 |
| Ciliary body involvement | 0.64 |
| Tumor base | 0.002 |
| Tumour width | 0.73 |
| Cell type | 0.94 |
| Scleral extension ( Pearson Chi-square test) | 0.01 |
