# Supplementary material for: Clinico-Pathological Association of Delineated miRNAs in Uveal Melanoma with Monosomy 3/Disomy 3 Chromosomal Aberrations
Source: PLoS One. 2016 Jan 26;11(1):e0146128. doi: 10.1371/journal.pone.0146128 (PMC4728065; doi:10.1371/journal.pone.0146128)
Supplement: S4 Table — (DOC) [file pone.0146128.s007.doc]

**S4 Table:** Correlation analysis of liver metastasis with clinico pathological parameters in UM patients under study

| Clinico pathological parameters  (Independent-t –test) | p-value |
| --- | --- |
| Chromosome 3 aberration | 0.282 |
| HSP27 | 0.90 |
| Age | 0.166 |
| Sex | 0.532 |
| Ciliary body involvement | 0.185 |
| Tumor base | 0.70 |
| Tumour width | 0.111 |
| Cell type | 0.165 |
