# Supplementary material for: Clinico-Pathological Association of Delineated miRNAs in Uveal Melanoma with Monosomy 3/Disomy 3 Chromosomal Aberrations
Source: PLoS One. 2016 Jan 26;11(1):e0146128. doi: 10.1371/journal.pone.0146128 (PMC4728065; doi:10.1371/journal.pone.0146128)
Supplement: S5 Table — (DOC) [file pone.0146128.s008.doc]

| **S5 Table:** List of top 10 up-regulated miRNAs obtained by ANOVA and SAM. | | | | | |
| --- | --- | --- | --- | --- | --- |
| S.No | List of top 10 up-regulated miRNAs obtained by ANOVA | | List of top 10 up-regulated miRNAs obtained by SAM | | |
|  | miRNAs | Fold change (Transformed ratio) | miRNAs | Score (d) | Fold change (Arbitrary units) |
|  | *hsa-miR-1238* | 7.8 | *hsa-miR-206* | 17.23 | 826.66 |
|  | *hsa-miR-191** | 7.7 | *hsa-miR-21** | 14.55 | 370.09 |
|  | *hsa-miR-498* | 6.4 | *hsa-miR-361-3p* | 14.44 | 137.55 |
|  | *hsa-miR-602* | 5.2 | *hsa-miR-378** | 14.33 | 263.02 |
|  | *hsa-miR-149** | 4.05 | *hsa-miR-101* | 13.80 | 314.705 |
|  | *hsa-miR-371-5p* | 3.9 | *hsa-miR-29c** | 13.60 | 250.02 |
|  | *hsa-miR-373** | 3.7 | *hsa-miR-532-3p* | 13.33 | 294.92 |
|  | *hsa-miR-623* | 3.7 | *hsa-miR-199a-5p* | 13.25 | 135.19 |
|  | *hsa-miR-296-5p* | 3.5 | *hsa-miR-497* | 13.23 | 248.17 |
|  | *hsa-miR-1268* | 3.26 | *hsa-miR-20b* | 12.46 | 141.35 |
