# Supplementary material for: Clinico-Pathological Association of Delineated miRNAs in Uveal Melanoma with Monosomy 3/Disomy 3 Chromosomal Aberrations
Source: PLoS One. 2016 Jan 26;11(1):e0146128. doi: 10.1371/journal.pone.0146128 (PMC4728065; doi:10.1371/journal.pone.0146128)
Supplement: S6 Table — (DOC) [file pone.0146128.s009.doc]

**S6 Table:** Clinico-pathological descriptions of the tumors mean and median fold change of individual miRNAs, statistical significance (p-value ≤ 0.05) derived between the clinico-pathological parameters and mean fold change of miRNAs expression.

| Clinico-pathological  parameter | No. of  samples | miR-214 | | | miR-let 7b | | | miR-149 | | | miR-143 | | |
| --- | --- | --- | --- | --- | --- | --- | --- | --- | --- | --- | --- | --- | --- |
|  |  | Median  RQ (IQR) | Mean  RQ (S.D.) | P-value | Median  RQ (IQR) | Mean  RQ (S.D.) | P-value | Median  RQ (IQR) | Mean  RQ (S.D.) | P-value | Median  RQ (IQR) | Mean  RQ (S.D.) | P-  value |
| Gender  Male  Female | 58  28 | 1.21(5.28)  0.60(6.68) | 0.94(4.38)  0.48 (3.6) | 0.63 | 2.32(5.75)  -2.27(7.85) | -3.04(4.50)  -2.57 (5.40) | 0.67 | 4.62(6.16)  4.81(10.34) | 4.91(6.18)  6.60 (6.48) | 0.25 | -0.80(7.30)  -0.22(6.44) | -1.27(6.06)  0.91(6.03) | 0.80 |
| Chromosome 3 aberration Monosomy  Disomy | 51  35 | 1.97(5.15)  -0.07(4.7) | 1.69(3.46)  -0.54(4.7) | 0.01 | -2.46(5.56)  -1.80(7.21) | -2.33(4.47)  -3.64(5.15) | 0.21 | 5.08(6.11)  4.13(5.81) | 5.75(6.69)  4.86(5.60) | 0.52 | 0.377(5.91)  -2.64(9.71) | -0.24(5.93)  -3.18(5.59) | 0.009 |
| CB Yes | 12 | 2.06(3.11) | 0.96(2.40) | 0.88 | -132(5.36) | -1.79(3.70) | 0.40 | 4.79(6.23) | 4.423(7.51) | 0.56 | 0.08(7.40) | -0.66(4.13) | 0.78 |
| No | 74 | 1.11(6.08) | 0.78(4.34) |  | -2.78(6.05) | -0.30(4.91) |  | 4.69(6.11) | 5.565(6.08) |  | -0.54(7.44) | -1.17 (6.28) |  |
| Tumour base (mm) <5mm | 3 | 0.93(0) | 1.13(1.46) | 0.55 | -1.40(0) | -1.40(0.42) | 0.92 | 3.97(0) | 4.19(3.0) | 0.78 | 0.60(0) | 0.12(3.81) | 0.85 |
| 6-8mm | 7 | 1.18(6.91) | 0.89(5.27) |  | -2.27(7.85) | -3.11(4.53) |  | 1.32(14.3) | 3.18(9.3) |  | -0.22(2.95) | -0.36(2.60) |  |
| 9-12mm | 34 | 0.11(3.75) | 0.03(4.35) |  | -2.83(6.35) | -3.14(4.48) |  | 5.20(6.75) | 7.97(6.63) |  | 0.74(10.44) | -0.67(8.10) |  |
| >12mm | 42 | 1.81(6.5) | 1.40(3.85) |  | -2.47(6.99) | -2.68(5.25) |  | 4.72(5.15) | 5.65(5.63) |  | -.83(6.38) | -1.67(4.42) |  |
| Tumour width (mm) <5mm | 12 | 2.30(6.80) | 1.27(5.01) | 0.77 | -3.17(7.22) | 3.68(4.96) | 0.50 | 1.74(6.43) | 1.22(5.02) | 0.01 | -0.26(4.61 | -0.87(4.63) | 0.84 |
| 6-8mm | 21 | 1.95(5.79) | 1.49(3.26) |  | -2.27(6.35) | -3.23(4.60) |  | 6.67(11.22) | 7.90(7.70) |  | -2.02(8.87) | -1.28(8.78) |  |
| 9-12mm | 32 | 1.27(4.68) | 0.43(4.43) |  | -2.32(6.48) | -2.84(5.01) |  | 4.53(7.54) | 6.13(5.99) |  | 0.92(7.71) | -0.48(5.47) |  |
| >12mm | 21 | 1.02(5.49) | 0.44(4.02) |  | -1.68(5.86) | -2.01(4.64) |  | 4.46(4.93) | 6.15(4.30) |  | -0.98(6.37) | -2.01(4.06) |  |
| Cell type  Spindle | 27 | 1.43(5.08) | 1.19(3.33) | 0.844 | -1.59(6.38) | -2.41(5.02) | 0.22 | 4.51(5.46) | 6.03(5.51) | 0.80 | 0.73(5.11) | -0.09(5.19) | 0.560 |
| Epitheliod | 15 | 1.25(6.88) | 0.62(4.01) |  | -2.94(6.11) | -4.14(4.57) |  | 4.46(5.47) | 4.80(5.43) |  | -0.82(8.48) | -1.88(5.55) |  |
| Mixed | 44 | 1.03(5.88) | 0.64(4.63) |  | -2.30(5.83) | -2.68(4.70) |  | 5.20(8.56) | 5.22(7.01) |  | -2.27(7.50) | -1.46(6.64) |  |
| Liver metastasis  Yes | 17 | 2.06(6.55) | 0.39(4.24) | 0.17 | -3.05(5.14) | -2.72 (4.80) | 0.44 | 6.49(10.88) | 4.73(6.05) | 0.04 | -1.72(8.36) | -0.90(6.38) | 0.37 |
| No | 69 | 1.02(4.96) | 1.94(3.59) |  | -2.28(6.31) | -3.73(5.00) |  | 4.51(5.88) | 8.16(6.98) |  | -0.30(7.61) | -2.37(4.75) |  |
| KM (2-5 years) | 52 | 22% |  |  | 71% |  |  | 5% |  |  | 39% |  |  |

| Clinico-pathological  parameter | No. of  samples | miR-134 | | | miR-146b | | | miR-1238 | | | miR-199a | | |
| --- | --- | --- | --- | --- | --- | --- | --- | --- | --- | --- | --- | --- | --- |
|  |  | Median  RQ (IQR) | Mean  RQ (S.D.) | P-value | Median  RQ (IQR) | Mean  RQ (S.D.) | P-value | Median  RQ (IQR) | Mean  RQ (S.D.) | P-value | Median  RQ (IQR) | Mean  RQ (S.D.) | P-value |
| Gender  Male  Female | 58  28 | 1.34(5.53)  2.01(5.86) | 1.64(4.41)  2.02(5.85) | 0.74 | 1.27(3.55)  2.10(5.76) | 0.60(5.35)  1.18(5.20) | 0.64 | 9.66(6.47)  7.46(6.65) | 9.301(4.59)  8.472(5.38) | 0.46 | 6.60(5.24)  7.10(8.52) | 6.59(5.59)  7.20 (6.32) | 0.65 |
| Chromosome 3 aberration  Monosomy  Disomy | 51  35 | 2.15(5.22)  0.61(7.46) | 2.63(4.27)  0.45(5.38) | 0.04 | 1.92(3.96)  0.79(7.64) | 1.99(3.63)  -1.00(6.70) | 0.009 | 9.32(6.35)  7.61(6.66) | 9.31(4.78)  8.5(4.93) | 0.45 | 7.34(5.58)  5.70(7.15) | 8.11(5.19)  4.78(6.14) | 0.009 |
| CB Involvement  Yes | 12 | 2.21(4.17) | 1.75(5.02) | 0.98 | 2.77(2.78) | 2.38(2.07) | 0.26 | 7.04(8.63) | 10.00(5.79) | 0.44 | 6.19(6.78) | 8.04(4.98) | 0.43 |
| No | 74 | 1.58(5.77) | 1.77 (4.8) |  | 1.43(4.96) | 0.55(5.56) |  | 9.04(5.82) | 8.83 (4.67) | 0.20 | 7.23(2.55) | 6.61(5.90) | 0.24 |
| Tumour base (mm)  <5mm | 3 | -2.90(0) | -2.37(1.81) | 0.28 | 0.60(0) | -3.50(8.2) | 0.47 | 0.26(0) | 2.35(4.12) | 0.08 | 7.29(0) | 7.27(5.96) | 0.53 |
| 6-8mm | 7 | 2.20(6.03) | 2.22(3.44) |  | 4.94(10.5) | 2.10(7.90) |  | 6.20(8.39) | 7.70(4.74) |  | 5.93(10.84) | 3.71(10.31) |  |
| 9-12mm | 34 | 0.99(5.66) | 1.13(5.41) |  | 1.37(3.33) | 1.07(4.17) |  | 8.20(6.57) | 9.44(4.52) |  | 6.19(4.67) | 6.93(4.81) |  |
| >12mm | 42 | 2.52(4.83) | 2.51(4.56) |  | 1.71(5.46) | 0.69(5.39) |  | 10.08(6.45) | 9.33(4.88) |  | 7.13(6.58) | 7.21(5.58) |  |
| Tumour width  <5mm | 12 | -1.24(7.23) | -0.65(5.62) | 0.44 | 0.57(7.75) | 0.42(6.40) | 0.41 | 7.44(6.43) | 8.32(3.59) | 0.90 | 9.2(10.36) | 6.58(3.88) | 0.44 |
| 6-8mm | 21 | 1.23(7.06) | 1.52(5.33) |  | 2.10(2.62) | 1.79(4.18) |  | 7.89(6.54) | 9.12(4.45) |  | 6.04(5.76) | 6.86(4.74) |  |
| 9-12mm | 32 | 2.48(5.20) | 2.75(4.59) |  | 2.39(5.25) | 1.11(4.05) |  | 9.36(5.38) | 8.80(5.13) |  | 7.69(5.35) | 7.89(5.86) |  |
| >12mm | 21 | 1.24(4.94) | 1.90(3.96) |  | 0.69(7.12) | -0.42(6.99) |  | 10.5(10.1) | 9.58(5.51) |  | 4.89(5.03) | 7.13(4.17) |  |
| Cell type  Spindle | 27 | 2.20(5.3) | 2.19(4.73) | 0.44 | 1.85(2.91) | 1.79(4.07) | 0.41 | 8.52(4.05) | 8.27(3.88) | 0.22 | 6.95(6.41) | 7.67(5.35) | 0.59 |
| Epitheliod | 15 | 0.01(4.04) | 0.32(5.07) |  | 0.07(4.05) | 0.36(3.90) |  | 7.61(6.0) | 7.74(4.73) |  | 6.96(7.0) | 7.06(5.27) |  |
| Mixed | 44 | .05(5.95) | 2.00(4.83) |  | 2.29(5.77) | 0.60(6.19) |  | 10.1(7.21) | 9.85(5.27) |  | 6.39(6.0) | 6.81(5.78) |  |
| Liver metastasis  Yes | 17 | 2.93(3.7) | 1.18(5.07) | 0.03 | 2.27(4.93) | 0.58(5.56) | 0.78 | 7.74(5.07) | 5.16  (0.64) | 0.65 | 8.34  (6.17) | 6.07(0. .75) | 0.53 |
| No | 69 | 1.14(5.4) | 4.03(3.5) |  | 1.46(4.3) | 0.99(4.1) |  | 9.31(7.16) | 3.61(0.87) |  | 6.40(5.58) | 5.13(1.24) |  |
| KM (2-5 years) | 52 | 13% |  |  | 13% |  |  | 2% |  |  | 7% |  |  |
| Y: Years, CB: Ciliary body involvement, KM : Kaplan-Meier metastasis –free survival analysis (Follow-up duration:2-5 years) | | | | | | | | | | | | | |
