# Supplementary material for: Clinico-Pathological Association of Delineated miRNAs in Uveal Melanoma with Monosomy 3/Disomy 3 Chromosomal Aberrations
Source: PLoS One. 2016 Jan 26;11(1):e0146128. doi: 10.1371/journal.pone.0146128 (PMC4728065; doi:10.1371/journal.pone.0146128)
Supplement: S8 Table — (DOC) [file pone.0146128.s011.doc]

**S8 Table: Downstream effect analysis of genes and miRNAs over-expressed in UM.**

| Biological function/pathway | Overlap p-value (Z-score) | Gene/miRNA | Prediction (based on expression direction) | Expression Disomy |
| --- | --- | --- | --- | --- |
| Primary Melanoma | 2.84E-07 | *hsa-mir-149* | Affected | 5.750 |
|  |  | *hsa-mir-146b* | Affected | 1.990 |
|  |  | *hsa-let-7b* | Affected | -2.330 |
| Metastatic melanoma cancer | 2.93E-06 | *hsa-mir-149* | Affected | 5.750 |
|  |  | *hsa-mir-146b* | Affected | 1.990 |
|  |  | *hsa-let-7b* | Affected | -2.330 |
| Proliferation of tumor cell lines | 4.21E-04 (1.621) | *hsa-mir-199a* | Increased | 8.110 |
|  |  | *hsa-mir-146b* | Decreased | 1.990 |
|  |  | *hsa-mir-21* | Increased | 1.690 |
|  |  | *hsa-mir-143* | Increased | -0.240 |
|  |  | *hsa-let-7b* | Increased | -2.330 |
| Apoptosis of tumor cell lines | 2.18E-03 (-1.304) | *hsa-mir-146b* | Increased | 1.990 |
|  |  | *hsa-mir-21* | Decreased | 1.690 |
|  |  | *hsa-mir-143* | Decreased | -0.240 |
|  |  | *hsa-let-7b* | Decreased | -2.330 |
| Proliferation of tumor cell lines | 9.79E-05 (0.811) | *HDAC8* | Increased | 3.520 |
|  |  | *KIT* | Increased | 1.820 |
|  |  | *WISP1* | Decreased | 0.500 |
|  |  | *SMAD4* | Increased | -0.640 |
