# Supplementary material for: Clinico-Pathological Association of Delineated miRNAs in Uveal Melanoma with Monosomy 3/Disomy 3 Chromosomal Aberrations
Source: PLoS One. 2016 Jan 26;11(1):e0146128. doi: 10.1371/journal.pone.0146128 (PMC4728065; doi:10.1371/journal.pone.0146128)
Supplement: S9 Table — (DOC) [file pone.0146128.s012.doc]

**S9 Table:**  mRNA expression analysis using qRT-PCR.

| S.No | Genes | Gene expression: Fold change in log _2_ ratio (S.D.) | |
| --- | --- | --- | --- |
|  |  | Monosomy (N=5) | Disomy (N=5) |
|  | *WISP1* | -1.16 (3.12) | 0.5 (2.32) |
|  | *HDAC8* | 3.81(2.34) | 3.52 (2.92) |
|  | *SMAD4* | -0.29(1.87) | -0.64(2.26) |
|  | *c-KIT* | 2.70(2.95) | 1.82(3.42) |
|  | *HIPK1* | 2.53(1.43) | 3.80(1.12) |
